# Genomic Characterization of External Morphology Traits in Kelpies Does Not Support Common Ancestry with the Australian Dingo

**DOI:** 10.3390/genes10050337

**Published:** 2019-05-03

**Authors:** Tracy Chew, Cali E. Willet, Bianca Haase, Claire M. Wade

**Affiliations:** 1 Sydney Informatics Hub, The University of Sydney, NSW 2006, Australia; tracy.chew@sydney.edu.au (T.C.); cali.willet@sydney.edu.au (C.E.W.); 2 Sydney School of Veterinary Science, The University of Sydney, NSW 2006, Australia; bianca.waud@sydney.edu.au; 3 School of Life and Environmental Sciences, the University of Sydney, NSW 2006, Australia

**Keywords:** coat-color, ear-type, Australian working Kelpie, Australian Kelpie, Dingo

## Abstract

The Kelpie is a breed developed in Australia for use as a livestock herding dog. It has been proposed that the development of the breed included gene flow from the Australian Dingo (*Canis dingo*), a canid species present on the Australian continent for around 4000 years. The Kelpie breed is split between working and conformation types that have readily recognizable differences in external morphology. We characterize known gene variants relating to external morphology in sequenced representatives of both Kelpie types (Australian Kelpie—conformation; Australian Working Kelpie—herding) and compare the variants present with those in sequenced Australian Dingoes, including 25 canids with locus-constrained data and one with a whole genome sequence. Variants assessed include identified coat color and ear morphology variants. We describe a new variant site in the transcribed region of methionine sulfoxide reductase 3 that may relate to ear phenotype. None of the morphology variants analyzed offer support for co-ancestry of the Kelpie breed with the Australian Dingo.

## 1. Introduction

The Kelpie breed is an iconic dog breed developed in Australia in the late 19th century. The Working Kelpie Council of Australia reports that the breed was developed from Scottish Smooth Collie and Farm Collie stock, and was founded upon a small number of key individuals. The breed is renowned for its resilience, working in harsh (hot, dry, and prickly) conditions in the Australian outback [1]. Since its inception the Kelpie population has become divided, as particular lines were developed for exceptional performance in specialist aspects of working dog performance. In addition, one group from the population was separated to participate in dog conformation showing. This breed division has resulted in readily visible phenotypic differences between the groups. For instance, there are marked coat color and patterning frequency differences between the working and conformation Kelpie types.

**Figure 1:**
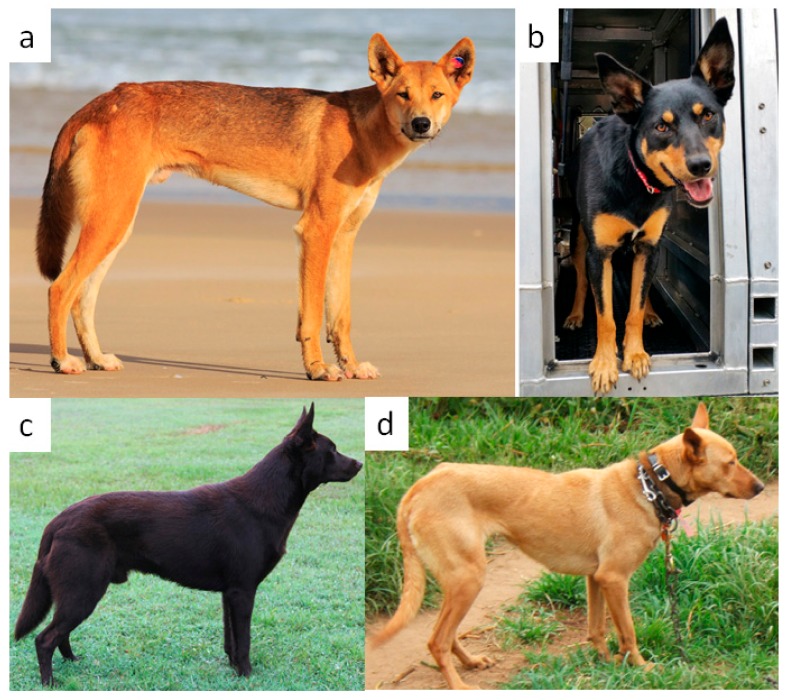
(**a**) Australian Dingo (*Canis dingo*), Fraser Island (image: IStockPhoto.com). (**b**) Australian Working Kelpie demonstrating tan patterning (image: Al Dodge Photography). (**c**) Australian Kelpie (image: Mandy Samson). (**d**) Ginger/Cream Australian Working Kelpie (image: Janelle Hansen).

The Australian Kelpie (AK) (Figure 1c) that has been bred for conformation and registered with the Australian National Kennel Council most often has a solid/self-color coat in either black or brown [1,2]. Homozygous coding variants and compound heterozygous genotypes coded by the gene *5*,*6-dihydroxyindole-2-carboxylic acid oxidase precursor* (*TYRP1*) may result in a brown color [3,4]. Solid black (self-color) may be coded by either a recessive variant at the *Agouti signaling protein* (*ASIP*) and *RNA-Binding Protein (Autoantigenic, Heterogeneous Nuclear Ribonucleoprotein-Associated With Lethal Yellow)* (*RALY*) loci or a dominant variant at the canine *β*-*defensin 103* (*CBD103*) locus [5]. Dogs that are genetically brown as a result of recessive alleles at *TYRP1* are observed to have varying pigment intensity, and the genes underlying this difference in hue are thus far undescribed in dogs with normal coat variation (as opposed to those affected by albinism) [4].

Australian Working Kelpies (AWK) (Figure 1b) registered with the Working Kelpie Council of Australia are bred for their livestock herding capability. Dogs of this variety are frequently taller and have longer bodies and muzzles than the AK type. These dogs typically exhibit the same two main base colors (black and brown), but frequently exhibit tan markings on the black or brown coat. Confusingly, dogs that are brown due to the activity of *TYRP1* are colloquially referred to as red in the breed. The tan markings are predicted to be due to alleles at either the *ASIP* locus (A-locus) or the *CBD103* locus (K-locus) [5,6]. The occurrence of the brindle phenotype encoded by *CBD103* [7] is undescribed in either variety of the Kelpie breed.

Other coat colors that exist in AWKs include yellow (known as *ginger* or *cream* in the breed), which can be driven by the pheomelanin variant at *Melanocortin-1 receptor* (*MC1R*) [4]. A red or yellow coat color may also be caused by the dominant yellow variant (A^y^) at *ASIP* [8]. Blue, fawn, and cream (all resulting from dilution at *Melanophilin* (*MLPH*) of black, brown, and ginger, respectively) [9,10] are observed in AWKs, and these may occur with or without tan markings.

White markings are rarely observed in the breed, and when present, typically result from recent out-crossing with other livestock herding breeds, such as the Border Collie. The white patterning in most dog breeds is controlled by variants in the melanocyte-specific promoter region of *Micropthalmia transcription factor* (*MITF*), which in combination impact the extent of white markings in the dog. The first variant in the region that is expected to impact the extent of white is a short-interspersed nuclear element (SINE) insertion three kilobases upstream of the melanocyte-specific promoter for the gene. The second variant associated with the extent of white is a length polymorphism in the immediate vicinity of the melanocyte-specific first exon [11]. In general, dogs homozygous for the SINE element are expected to have either piebald or extreme white markings. At the length polymorphism, dogs with longer alleles are expected to have some white markings. The alleles behave in a co-dominant manner, with intermediate white markings observed on heterozygotes of the variant allele types.

Throughout the history of the Kelpie breed, speculation has persisted that the Australian wild dog *Canis dingo* (Dingo) contributed to its founding. Such ideas were likely fueled by statements attesting this by respected individuals such as geneticist Dr. R. B. Kelley, the author of a widely recognized book used in breeding and training livestock herding dogs first published in 1942 [12]; more recently, others suggest, albeit without peer review, that DNA demonstrates common ancestry with the Dingo [13]. Others who claim personal knowledge of the breed founding lines [14], or who report that attempted crosses between Kelpies and Dingoes to improve working traits failed regardless of the high quality of the Kelpies used in the crossing [15], offer evidence to the contrary.

The Dingo (Figure 1a) has an external phenotype that is quite like the Kelpie. The two dog types are of similar size (the Dingo may be slightly larger). Both have erect ears. While Dingoes are most commonly yellow-gold in color as adults, they can occur with dark coat colors on occasion. Both dog types have similar hair length and texture. Many laypeople believe that the ginger and cream variants in the Kelpie breed (Figure 1d) are derived from the Dingo ancestral type. In common with the Dingo, both breed varieties most commonly exhibit erect ears (pricked as opposed to drop-ears). While ears with complete drop are very rare in the Kelpie, some individuals have ears that are incompletely erect. Segregation of single nucleotide polymorphism (SNP) markers with the ear phenotype has been considered extensively in the literature, although no functional mutation has been proposed [16,17]. The gene *Methionine sulfoxide reductase 3* (*MSRB3*) has been suggested as a strong regional candidate gene for the phenotype [17].

Our analysis will determine the mutational basis of commonly observed external morphologies (coat color and ear phenotype) in the two Kelpie breed varieties at loci for which variants have been described. We include a description of two insertion variants in the 3′ untranslated region (UTR) of the gene *MSRB3* that have not been reported in the literature. Both longer variants in the *MSRB3* UTR are in near complete linkage disequilibrium, with a potentially functional array SNP that has been strongly associated with prick and drop ears in many breeds. None of the analyzed variants support co-ancestry with the Australian Dingo.

## 2. Materials and Methods

### 2.1. Samples and Sequencing

Genomic DNA was extracted from 12 individual EDTA-stabilized whole blood samples (one Australian Kelpie (USCF305)-conformation-type; eight AWKs (USCF634, USCF635, USCF636, USCF639, USCF640, USCF6348, USCF6350, USCF6359), and three Kelpies of undocumented type (USCF6182, USCF6203, USCF6343), using the Illustra Nucleon BACC2 kit (GE Healthcare, Pittsburgh, CA, USA). DNA was provided to the Ramaciotti Centre (University of New South Wales, Kensington, Australia) for whole-genome next-generation sequencing. Next-generation sequencing was performed on the Illumina HiSeq 2000 or HiSeq 2500 (Illumina, San Diego, CA, USA) as 100 base-pair paired end reads on a single lane of the sequencing platform with Tru-seq library preparation (Illumina) [1,18].

Sequence data for the Labrador retriever LA882 (Accession identifier: ERX425617) and the Dingo (Accession identifier: RKW13760) were obtained from the Sequence Read Archive (SRA) in Genbank (https://www.ncbi.nlm.nih.gov/sra/) [19]. The Dingo was sourced from the Bargo Dingo Sanctuary, and was reported to demonstrate no genetic evidence of hybridization based on a marker panel.

Locus-constrained sequencing data for the *ASIP*, *CBD103*, and *MC1R* loci were available for 25 Dingoes in the public domain [20].

### 2.2. Ethics

Recommendations from the Australian Code for the Care and Use of Animals for Scientific Purposes were strictly adhered to throughout this study. Research was conducted with animal ethics approval, granted by the Animal Ethics Committee at the University of Sydney (approval number N00/9–2009/3/5109, 24 September 2009).

### 2.3. Bioinformatics

Raw reads were aligned as pairs to the CanFam 3.1 reference sequence using the Burrows–Wheeler Alignment-MEM tool (version 0.7.15) with default parameters [21,22]. Polymerase chain reaction (PCR) duplicates were marked with SAMBLASTER (version 0.1.22) [23]. Local realignment was performed around insertion–deletions, and base quality scores were recalibrated with the Genome Analysis Tool Kit (GATK, version 3.6.0) [24]. Indexing of the reference sequence and alignment (BAM) files was performed with SAMtools (version 0.1.19) [21].

### 2.4. Variants

The variants previously identified as coding for the external morphologies used as breed descriptors in the Kelpie breed standards [25] for ear type and coat color were selected from those nominated in Online Mendelian Inheritance in Animals (OMIA) for the relevant phenes [26] (Table 1). Where genomic location was unidentified in OMIA, we used visual inspection of the DNA sequence in the OMIA described region as ascertained by in silico PCR (http://genome.ucsc.edu), using primers described in the relevant article reporting the variant or our own variant calling algorithms to identify the location of the variant. Locus labelling (A-, B-, D-, E-, and S-) is based on nomenclature described in [27], other than the K-locus, which was described in Candille et al. [5].

The coat color variants explored were those known to occur at the A-loci (*ASIP* and *RALY*) [6,8,28], the B-locus (*TYRP1*) [4], the K-locus (*CBD103*) [5], the E-locus (*MC1R*) [4,29], and the S-locus (*MITF*) [11,30]. The S-locus is the major locus driving the occurrence of white markings. We also considered the D-locus (dilution), considering the gene *MLPH* that affects dilution of the base coat color [10].

The SNP variant used to assess ear morphology [16] was re-mapped onto the Canfam 3.1 reference sequence using University of California Santa Cruz Lift Over tool (http://genome.ucsc.edu). Manual inspection of sequence alignment throughout the region of *MSRB3* was used in conjunction with variant calling (four reads score of 10-base quality) per allele required) from the whole genome sequence, in order to identify a new variant that segregates in strong linkage disequilibrium with the prick versus drop ear phenotype in the sequenced cohort. This variant was compared among the sequenced Kelpies of both varieties; the genome reference (Boxer breed, expected to be homozygous for drop ear); a publicly available Labrador sequence, representing a validation of the drop ear variant [31]; and a publicly available Dingo sequence [19].

Further locus-constrained sequencing data from 25 Dingoes for exon 4 of *ASIP*, the coding region of *MC1R*, and the coding region of *CBD103* were available in Genbank [20]. These were aligned using Clustal–Omega [32] and the variants were manually assessed.

## 3. Results

### 3.1. Coat Color

Observed variants at the major coat loci impacting the Kelpie breed varieties, based on whole genome sequencing data, are shown in Table 2. The stop codon variant at *MC1R* that is known to prevent the expression of eumelanin, thus generating a yellow-red coat hue, was observed as a heterozygote in both Kelpie varieties (AK represented by USCF305, and AWK represented by USCF634, USCF635, USCF636, USCF639, USCF640, USCF6348, USCF6350, and USCF6359), but only one of 26 sequenced Dingoes had this variant as a heterozygote. The *MC1R* variant identified as causative for melanistic masking segregated freely in the working Kelpies as g.63694460_C>T (reference is C, which is masked) [29].

The two Kelpies that were known to be brown in the data (AK USCF305 and AWK USCF6059) were homozygous for the *TYRP1* mutation at CFA11 g.33326685, p.331Q>X (described as “Dove” by Schmutz et al. [4]). No further *TYRP1* alleles predicted to result in brown coat color were observed in the sequenced animals. Brown has not been officially recorded in the Dingo to date, to the authors’ knowledge.

At *MITF*, the prevalent length polymorphism allele observed in both varieties of Kelpie is one that is as yet undescribed [11,30]. The sequence is 10C9A2G12A, which might be labelled as 33C when using the nomenclature of previous authors. Allele labels are typically annotated by the variant length, and then awarded an alphabetical letter relating to their order of discovery. At the *MITF* length polymorphism, the variant alleles are encoded in a concise idiosyncratic gapped alignment report (CIGAR) format that is used in the standard sequence alignment map (SAM) format. For example, the canine reference genome allele is termed as 35A, and the variant can be annotated as: “12C9A2G12A”, which corresponds with the sequence “CCCCCCCCCCCCAAAAAAAAA GGAAAAAAAAAAAA” [11]. One dog (USCF640) was heterozygous for allele 31A [30]. All Kelpies were negative for the SINE insertion polymorphism at g.21836232_21836427 (Table 2) [11].

Tan markings were expected to be affected by either the A-loci (*RALY* or *ASIP*) or the K-locus (*CBD103*). The dominant black allele (*CBD103* CFA16, g.58965449-58965451ins>del) [5] was observed to be present, and explained the solid coloration in AK USCF305 (conformation), which was homozygous for the deletion allele. All sequenced Australian Working Kelpies were homozygous for the reference allele (yellow-variant) at *CBD103*. Of the 25 Kelpies previously sequenced, 24 were homozygous for the reference allele (ins/ins) and one was heterozygous [20]. The whole-genome-sequenced Dingo had the reference allele (Table 2).

All sequenced Kelpies (both AWK and AK) were homozygous at the *ASIP* locus for A^y^ CFA24:23393510_G (reference is T), CFA24:23393514_G (reference is A), and CFA24:23393552_G (reference is G). These alleles correspond with wild-type at the *ASIP* exonic locus (A82, R83, R92) [8]. Previous sequencing [20] of the fourth exon of *ASIP* demonstrates that among 22 Australian dingo sequences derived from geographically dispersed locations, 16 were homozygous for the A^y^ allele, 6 were heterozygous for wild-type, and one was homozygous for the wild-type variant. The Dingo with whole genome sequence was homozygous for the A^y^ variant (Table 2). Sequences were not available for the SINE-insertion allele that differentiates the wild-type from the black and tan phenotype. An *ASIP* coding variant that leads to recessive black was unobserved in any of the animals analyzed, and unobserved among dingoes in the previous sequencing [20].

One sequenced AWK (USCF640) was observed to carry the known *MLPH* mutation [10], but this was observed on a single sequencing read, and so we were unable to assign a complete genotype. Other dogs had poor genomic coverage over the region of the mutation. This is a sequencing artefact resulting from high GC nucleotide content in the vicinity related to the proximity of the mutation to the first exon of the gene.

Five of the Kelpies with whole genome sequencing had no coat color reported in their sample file. From the results of the current analysis, we can predict the colors of three dogs (USCF6182, USCF6203, and USCF6350) to be brown with tan points; USCF6343 is likely black with tan points (carrying red/yellow—the melanistic mask from *MC1R* will be invisible on this coat background); and USCF6348 is likely black with tan points (carrying brown at *TYRP1*).

### 3.2. Ear Morphology

Erect ears are the most frequently observed ear phenotype in both Kelpie varieties. By sequencing, AK (USCF305), and AWK individuals (USCF635, USCF636, USCF639, USCF640, USCF6348, USCF6350, USCF6359) are predicted to have erect ears, as they are homozygous for the non-reference allele at the putative locus (CFA10 g.8085469C>T, Table 3), located in the intergenic region between *MSRB3* and *High Mobility Group AT-Hook 2 (HMGA2)* [17]. AWK USCF634 may have semi-erect ears, according to its heterozygous genotype at CFA10 g.8085469C>T.

A new possible functional variant in *MSRB3* is identified in this study (g. 8038433del>ins). At this locus, the Dingo has a 5-base insertion variant (g. 8038433del>insTTTAT). All Kelpies have a 10 bp insertion at the same locus (g. 8038433del>insTTTATTTTAT). The AWK dog (USCF634) that was heterozygous at the intergenic SNP locus had insufficient sequencing coverage at the site of the insertion to exclude the possibility of the dog also being heterozygous at that locus.

### 3.3. Morphologic Concordance of Kelpie with Dingo

There is little or no evidence in the present data to support a history of significant introgression of Dingo into either Kelpie variety. The Dingo and domestic Kelpie populations have different segregation of yellow/ginger coat color (Kelpie via *MC1R* while Dingo via *ASIP*) (Table 2). The populations also have different alleles at *MITF*, *RALY*, and *MSRB3*. The Dingo variant at *MITF* (11C10A2G12A) has been previously described as being observed in a Scandinavian wolf.

Kelpies are predicted to have erect ears (CFA10 g.8085469C>T (T/T)). They also carry a 10-base insertion variant: *MSRB_UTR* g. 8038433del>insTTTATTTTAT. The Dingo is predicted to have erect ears by both loci (CFA10 g.8085469C>T (T/T), *MSRB3_UTR* CFA10 g. 8038433del>insTTTAT (ins/ins)), while the Labrador is predicted to have drop ears by both loci (C/C and del/del respectively).

Accession identifiers associated with the resource are listed in Supplementary Table S1. The project PRJEB28163 is housed in the European Nucleotide Archive (https://www.ebi.ac.uk/ena/data/search?query=PRJEB28163).

## 4. Discussion

Despite the commonly held view that the Dingo is an ancestral contributor to the Australian Kelpie [12,13,15], there is scant evidence in the current sequencing data to support this assertion. It is predominantly the morphologic similarities between the Dingo and Kelpie varieties that underlie the conjecture of introgression of Dingo into the original Kelpies. Here, we observe that different alleles segregate in the domestic and wild canids at the loci underpinning the same external morphologies that on the surface appear to be shared between the Dingo and the two Kelpie varieties.

The A-loci (*ASIP-RALY* on chromosome 24) underlie solid color (black/brown/yellow) and *saddle* or *tan-point* coat markings. At the *ASIP*-locus, most wolves would have the wild-type allele, whilst dingoes and many domestic dogs have other variants. Kelpies and Dingoes are strongly diverged at the A-loci (*ASIP-RALY* on chromosome 24), where in Dingoes the dominant yellow (A^y^) haplotype has the highest frequency in conjunction with a variant at the nearby *RALY* gene, supporting a “saddle” pattern of markings. At these loci, the Kelpie exclusively has a haplotype that supports the non-agouti phenotype, with *tan-point* markings (a^t^) at the *RALY* locus and *wild-type* variants in *ASIP*. While the *saddle* pattern from *RALY* is not readily visible in the Dingo, this is likely affected by the A^y^ haplotype at *ASIP*.

Ginger/yellow in the Kelpie is likely caused by the recessive CFA5 g.63694334G>A variant coded by the gene *MC1R*, as this allele was observed in the heterozygous state in 3 of 12 sequenced Kelpies (both varieties). In the Dingo, yellow coat color is driven by the A^y^ allele at the *ASIP* locus and homozygosity for the insertion variant at *CBD103* (reference allele), which enables colors encoded by variants at the A-loci (*RALY-ASIP*) and E-locus (*MC1R*) to be observed [5]. Among 26 Dingoes (including 25 Dingoes sequenced previously for the *MC1R* coding region), only one was heterozygous for the *MC1R* CFA5 g.63694334G>A variant (allele frequency <2%) [20].

*CBD103 dominant black* (del/-) is perhaps better described as self-color, as it is epistatic to the genotype at *ASIP*, where the phenotype is black in the presence of homozygosity for the non-agouti allele (most usually a^t^) at the A-locus [5]. The representative sequenced Dingo in this study was homozygous for the insertion (pheomelanin expressing) allele of this gene. Among 25 Dingoes sequenced for the coding portion of this gene, all were homozygous for the insertion variant, but one was equivocal and possibly heterozygous [20].

Among Kelpies, the major coat color difference between the two assessed varieties is the presence or absence of tan markings. We show that this variation is controlled by the *CBD103* gene (commonly known as the K-locus) [5]. Both Kelpie breed varieties are concordant (homozygous wild-type) at *ASIP*, which has been proposed in the past to control black versus black and tan. Both Kelpie varieties actively segregate *TYRP1* and *MC1R*-red, although ginger/cream are disallowed by the Australian National Kennel Council breed standard, which affects the AK variety. Melanistic mask was observed to freely segregate in the AWK. The only observed coding variant at *TYRP1* is p.Q331X, and this is the same coding variant for brown coat color for both AK and AWK groups. Differences in pigment intensity or hue in brown kelpies remain unexplained by the loci described thus far. The Dingo RKW13760 was homozygous for the black eumelanin expressing variant at *TYRP1*.

Dilution as coded by *MLPH* was not assessed thoroughly in this analysis, due to poor sequence quality caused by high local G–C nucleotide content. One dog with low-quality cover (AWK USCF640) had a single sequencing read that is concordant with the dog being heterozygous for the presence of the MLPH splicing variant. Dilute animals are known to be present within the variety.

The length polymorphism in the melanocyte-specific promoter of *MITF* defines a spectrum of solid to spotted coat markings in dogs. The allele that is most commonly observed in both Kelpie varieties is yet undescribed in the literature (length of 33 bases, coded by 10C9A2G12A). The absence of the SINE element CFA20 g.21836232_21836427ins>del at the *MITF* locus would suggest that if white markings were to occur in either Kelpie variety, they would be of modest extent. The allele observed at *MITF* in the Dingo has been previously described as *wild-type* 35B (observed in a Scandinavian wolf) [30]. The sequenced Dingo was additionally homozygous for the SINE element insertion at CFA20 g.21836232_21836427, which is predictive of the presence of piebald or extreme white markings. The SINE element insertion and others of the longer haplotypes at CFA20 g.21839321_21839366 (indel) have been commented upon [30], because the lengths of the alleles would predict the presence of white markings, which are rare in wolves. However, it is not uncommon for Dingoes to have white paws and chest. It is possible that alleles at *MITF* are epistatic with those at the A-locus, to influence the penetrance of white markings as coded by the *MITF* loci.

Erect ears are common between the Dingo and both Kelpie varieties. The SNP variant [17] detected in the Kelpie at the ear locus concurs with that of the Dingo; however, the insertion allele in the nearby MSRB3_UTR (this study) differs between the domestic and wild dogs, suggesting that the variants have no recent co-ancestry. Other aspects of ear structure than ear erectness are as yet unpublished in the canine scientific literature.

This study is limited by the genetics observable in modern-day Kelpies. It is possible that attempts at introgression of the Dingo into the Kelpie occurred historically, but that the Dingo genes did not persist in the population at the loci observed. The possible presence of the dominant black *CBD103* allele in one sequenced Dingo may suggest introgression in that canid, or may represent a natural low frequency variant in that population. In either case, that animal would be expected to have a black coloration. Further investigation of the *MC1R* sequence for this animal (Alpine5) reveals a likely coding variant near the start codon for the gene, which may result in a gold coloration for this animal (Accession KF586907.1).

## 5. Conclusions

Examination of morphological variants assayed in representative sequenced *C. dingo* and 12 dogs designated as Kelpies (varieties: Australian Kelpie and Australian Working Kelpie) failed to support the assertion of significant historical introgression of Dingo genetics that is commonly described in Australian folklore. While the external phenotypes of the animals can be quite similar (Figure 1), particularly in Kelpies that are homozygous for red–yellow variants of *MC1R* (Figure 1d), there is little evidence that these external physical similarities have the same underlying genetic causes. The domestic and wild canids were strongly diverged at *MITF*, *MC1R*, *RALY*, and *MSRB3*. The two strains or varieties of Kelpie are similar at most loci, but are diverged at the K-locus (*CBD103*), explaining their difference in coat patterning.

## Figures and Tables

**Table 1:** Online Mendelian Inheritance in Animals (OMIA) phenes.

Phene	Gene	OMIA Code
Coat color, agouti	*ASIP*	000201-9615
Coat color, brown	*TYRP1*	001249-9615
Coat color, dilute	*MLPH*	000031-9615
Coat color, dominant black	*CBD103*	001416-9613
Coat color, extension	*MC1R*	001199-9615
Coat color, grizzle	*MC1R*	001495-9615
Coat color, melanistic mask	*MC1R*	001590-9615
Coat color, saddle tan vs. black-and-tan	*RALY*	001806-9615
Coat color, white spotting	*MITF*	000214-9615
Ears, folded	-	000319-9615

**Table 2:** Observed segregation of major coat color variants in 12 Kelpies and one Dingo. Owner-declared coat colors in parentheses.

Chromosome	Gene Name	Canfam 3.1 Reference Allele	Position in CanFam3.1 (Reference >Alternate)	*Canis dingo*_RKW13760	^‡^ AK-USCF305 (Brown)	USCF6182	USCF6203	^ƛ^ AWK-USCF634 (Black and Tan)	USCF6343	AWK-USCF635 (Black and Tan)	AWK-USCF636 (Black and Tan)	AWK-USCF639 (Black and Tan)	AWK-USCF640 (Black and Tan)	AWK-USCF6348	AWK-USCF6350	AWK-USCF6359 (Brown and Tan)	Variant Phenotype
5	*MC1R*	G	g.63694334G>A	G G	G A	G G	0 0	G A	G A	G G	0 0 ^†^	G G	G G	G G	G G	G G	Ginger (p.R306*) (recessive) (AA)
5	*MC1R*	C	g.63694460C>T	T T	T T	T T	T T	C T	T T	C C	T T	C C	C C	T T	T T	T T	Melanistic mask (p.M264V)(C -)
11	*TYRP1*	T	g.33317810T>C	T T	T T	T T	T T	T T	T T	T T	T T	T T	T T	T T	T T	T T	Brown (p.C41S)(recessive) (C C)
11	*TYRP1*	T	g.33319349T>G	T T	T T	T T	T T	T T	T T	T T	T T	T T	T T	T T	T T	T T	Brown (p.Tyr185*) (recessive) (G G)
11	*TYRP1*	C	g.33326685C>T	C C	T T	T T	T T	C C	C C	C T	C T	0 0	C C	C T	T T	T T	Brown (p.Q331X) (recessive) (T T)
11	*TYRP1*	ins	g.33326727 _33326729ins>del	ins/ins	ins/ins	ins/ins	ins/ins	ins/ins	ins/ins	ins/ins	ins/ins	ins/ins	ins/ins	ins/ins	ins/ins	ins/ins	Brown (p.345delP) (recessive) (del del)
16	*CBD103*	ins	g.58965449 _58965451ins>del	ins/ins	del/del	ins/ins	ins/ins	ins/ins	ins/ins	ins/ins	ins/ins	ins/ins	ins/ins	ins/ins	ins/ins	ins/ins	Black (dominant) (del -)
20	*MITF*	ins	g.21836232 _21836427ins>del	ins/ins	del/del	del/del	del/del	del/del	del/del	del/del	del/del	del/del	del/del	del/del	del/del	del/del	SINE element insertion is associated with extreme white spotting (recessive)
20	*MITF*	12C9A2G12A	g.21839321 _21839366 (indel)	11C10A2G12A/11C10A2G12A	10C9A2G12A/10C9A2G12A	10C9A2G12A/10C9A2G12A	10C9A2G12A/10C9A2G12A	10C9A2G12A/10C9A2G12A	10C9A2G12A/10C9A2G12A	10C9A2G12A/10C9A2G12A	10C9A2G12A/10C9A2G12A	10C9A2G12A/10C9A2G12A	10C8A2G11A/10C9A2G12A	10C9A2G12A/10C9A2G12A	10C9A2G12A/10C9A2G12A	10C9A2G12A/10C9A2G12A	Various. Longer variants are associated with more white markings
24	*RALY*	del	g.23252754 _23252770dupCCCCAGGTCAGAGTTT	del/del	ins/ins	ins/ins	ins/ins	ins/ins	ins/ins	ins/ins	ins/ins	ins/ins	ins/ins	ins/ins	ins/ins	ins/ins	as (del -)/at (ins/ins)
24	*ASIP*	del	g.23365298ins239	del/del	ins/ins	ins/ins	ins/ins	ins/ins	ins/ins	ins/ins	ins/ins	ins/ins	ins/ins	ins/ins	ins/ins	ins/ins	Ay (del -)/at (ins/ins)
24	*ASIP*	T	g.23393510T>G	T T	G G	G G	G G	G G	G G	G G	G G	G G	G G	G G	G G	G G	Sable/Fawn (Ay is T -)
24	*ASIP*	A	g.23393514A>G	A A	G G	G G	G G	G G	G G	G G	G G	G G	G G	G G	G G	G G	Sable/Fawn (Ay is A -)
24	*ASIP*	C	g.23393552C>T	C C	C C	C C	C C	C C	C C	C C	C C	C C	C C	C C	C C	C C	Black (recessive) (a is T T)

^†^ 0 0 no genotype call; ^‡^ AK Australian Kelpie; ^ƛ^ AWK Australian Working Kelpie.

**Table 3:** Segregation of an insertion polymorphism in the functional candidate gene *MSRB3* 3’ untranslated region (*MSRB3*_UTR) with pricked ear in 12 Kelpies, one Dingo, and one Labrador retriever.

Chromosome	*Gene Name*	Canfam 3.1 Reference Allele	Position in CanFam3.1 CanFam3.1 (Reference>Alternate)	Labrador_LA882 (Drop)	*Canis dingo*_RKW13760 (Prick)	^‡^ AK-USCF305 (Prick)	^ƛ^ AWK-USCF634	AWK-USCF635	AWK-USCF636	AWK-USCF639	AWK-USCF640	AWK-USCF6348	AWK-USCF6350	AWK-USCF6359 (Prick)	USCF6182	USCF6203	USCF6343	Variant Phenotype
10	*MSRB3_UTR*	del	g. 8038433del>insTTTATTTTAT g. 8038433del>insTTTAT	del/	insTTTAT/	ins/	ins/	ins/	ins/	ins/	ins/	ins/	ins/	ins/	ins/	ins/	ins/	Drop ears are del/del
				del	insTTTAT	ins	ins ^†^	ins	ins	ins	ins	ins	ins	ins	ins	ins	ins	
10	*Intergenic (MSRB3-HMGA2)*	C	g.8085469C>T	C C	T T	T T	T C	T T	T T	T T	T T	T T	T T	T T	T T	T T	T T	Drop ears are C C

^†^ Low sequence coverage does not exclude heterozygosity. ^‡^ AK: Australian Kelpie. ^ƛ^ AWK: Australian Working Kelpie.

## Supplementary Materials

The following are available online at https://www.mdpi.com/2073-4425/10/5/337/s1, Table S1: Accession numbers in the European Nucleotide Archive.
